# Injury Profile in German Amateur Women’s Football Is Knee-Centred and Match-Dominated: A Prospective One-Season Cohort Study

**DOI:** 10.3390/jcm15135059

**Published:** 2026-06-29

**Authors:** Niklas Engel, Markus Geßlein, Maximilian Willauschus, Andreas Kopf, Lotta Hielscher, Lorenz Huber, Hermann Josef Bail, Michael Millrose, Werner Krutsch, Michael Zalmanovici Trestioreanu, Johannes Rüther

**Affiliations:** 1 Department of Orthopedics and Traumatology, Paracelsus Medical University, 90471 Nuremberg, Germany; 2 FIFA Medical Centre of Excellence, University Medical Centre Regensburg, 93053 Regensburg, Germany; 3 Department of Trauma Surgery, University Medical Centre Regensburg, Franz-Josef-Strauss-Allee 11, 93053 Regensburg, Germany; 4 Department of Trauma Surgery and Sports Medicine, Garmisch-Partenkirchen Medical Center, 82467 Garmisch-Partenkirchen, Germany; 5 SportDocsFranken, 90471 Nuremberg, Germany; 6 Spitalul Clinic de Ortopedie, Traumatologie și Tuberculoză Osteoarticulară “Foișor”, 030167 Bucharest, Romania

**Keywords:** women’s football, injury epidemiology, amateur football, anterior cruciate ligament, injury mechanism, prospective study

## Abstract

**Background:** Injury epidemiology in women’s football has primarily focused on elite athletes, whereas the amateur level, representing the vast majority of female players worldwide, remains largely under-explored. We aimed to provide a first prospective one-season characterisation of injuries in German amateur women’s football. **Methods:** In this prospective observational cohort study, 26 clubs comprising 450 female amateur players in Bavaria were monitored over the 2023/24 season using standardised time-loss injury questionnaires as used in current professional registry studies. Injuries were characterised by anatomical location, type, mechanism, and activity context (match vs. training); proportions are reported with 95% binomial exact confidence intervals (CI). **Results:** A total of 68 injuries were recorded. Regarding injuries with documented information, 66.7% (95% CI 54.0–77.8) were acute traumatic and 78.2% (95% CI 65.0–88.2) occurred during matches. Lower-extremity injuries dominated (80.3%; 95% CI 71.2–90.5), led by the knee as the single most frequent region (28.2%; 95% CI 18.1–40.1). Ligament injuries were the most common injury type (27.9%). Seven ACL ruptures were documented (10.3%; 95% CI 4.2–20.1), all of which occurred during match play. Indirect-contact mechanisms were significantly over-represented among all knee injuries (OR 6.92, 95% CI 1.52–31.6; *p* = 0.012). **Conclusions:** Amateur injury profile in this study reproduced the knee-centred, match-dominated pattern of elite female football while carrying a relative ACL burden at the upper end of professional reference data; given the small number of ACL events (*n* = 7), this signal is hypothesis-generating. These findings support the extension of structured neuromuscular prevention programmes into German amateur women’s football.

## 1. Introduction

Women’s football has experienced substantial growth in participation over recent decades, creating an urgent need for sex-specific epidemiological data to inform prevention and medical management at all levels of the game [1,2]. Yet the available evidence is heavily skewed towards the elite level, while the amateur game—where most of the female players train and compete—remains largely under-explored.

The existing literature consistently identifies sprains, contusions, and muscle strains as the most common injuries among female players [1,2]. Studies in UEFA elite women’s competitions and the English Women’s Super League report comparable profiles, with lower-extremity injuries accounting for 66–87% of all events, predominantly affecting the knee, ankle, and thigh [3,4,5,6]. Gaulrapp et al. specifically identified the knee (31.0%) and ankle (22.1%) as the most frequently injured anatomical regions in the German Women’s Bundesliga, followed by the thigh (12.9%) and head (7.1%) [1]. In Germany, recent registry research studies by the FIFA Medical Centre Regensburg have begun to systematically capture injuries and illnesses in the men’s and women’s 1st and 2nd Bundesliga, providing valuable reference data for the professional level [7]. Additional reference data on professional German female football are provided by the German Statutory Accident Insurance for the administrative sector (Verwaltungs-Berufsgenossenschaft, VBG), Sports Reports [8].

Regarding injury context, Jacobson and Tegner reported that approximately 60% of injuries in elite female players occurred during matches [9]. Gaulrapp et al. observed an overall injury rate of 3.3 per 1000 h of exposure, with match-related risk more than ten times higher than training risk (18.5 vs. 1.4 per 1000 h) [1]. These patterns have been corroborated by multiple subsequent prospective studies [2,6,10].

Anterior cruciate ligament (ACL) injuries represent a particular concern in women’s football. Female players face a two- to threefold higher risk of ACL injury compared to their male counterparts [11,12,13], with a weekly knee injury prevalence of 4.3% reported among elite women [14]. Walden et al. reported an ACL injury incidence of 0.31 per 1000 h of exposure in adolescent female players [15], while Ekstrand et al. identified an overall rate of 20.6 ACL injuries per 1000 match hours in elite European women’s football [3]. Systematic video analyses have identified distinct injury mechanisms during cutting, landing, and pressing situations in women’s professional football [16]. Recognised risk factors include neuromuscular control deficits, biomechanical loading patterns, and hormonal influences across the menstrual cycle [17]. This elevated risk is multifactorial: anatomical and developmental characteristics (a narrower intercondylar notch, greater ligamentous laxity, and altered hamstring recruitment), hormonal fluctuations across the menstrual cycle, and neuromuscular and biomechanical loading during cutting, landing, and pressing all contribute, and meta-analytic data confirm an approximately two-fold higher ACL incidence rate in women that is independent of playing level [18,19,20]. Extrinsic and contextual factors—including disparities in access to strength and conditioning, pitch quality, and equipment—may further elevate risk and are particularly pronounced in the amateur game [18]. Further, recent German data demonstrated that the absolute risk of ACL rupture is even higher in amateur than in professional football and increased further after the COVID-19 lockdown period [21,22].

Despite this growing body of research, most studies focus on elite athletes. Indeed, a recent meta-analysis of prospective surveillance in senior women’s football identified only two amateur-club studies, and heterogeneous injury definitions precluded pooling of their injury rates [2], underscoring how sparse high-quality amateur data remain. Amateur football presents distinct challenges: limited medical resources, heterogeneous training routines, and difficulties in systematically tracking match and training exposure [22,23]. Understanding injury patterns at the amateur level is essential for developing effective, accessible prevention strategies applicable to the largest segment of the female football population.

The present study provides a first prospective one-season report of injuries in German amateur women’s football. The primary aim was to characterise the frequency, type, anatomical location, mechanism, and activity context (match vs. training) of injuries sustained by female amateur football players in Bavaria during the 2023/24 season. As a secondary aim, we hypothesised that the amateur injury profile would reproduce the lower-extremity, knee-centred and match-dominated pattern established in elite female football, while exhibiting a relative anterior cruciate ligament (ACL) burden at least as high as that reported in professional cohorts.

## 2. Materials and Methods

### 2.1. Study Design

This was a prospective observational cohort study conducted across 26 women’s amateur football clubs in Bavaria, Germany, spanning the full 2023/24 competitive season (August/September 2023 to June/July 2024). The study was approved by the Ethics Committee of the University of Regensburg (ethics approval number: 22-2919_2-101) and conducted in accordance with the Declaration of Helsinki. All participants provided written informed consent before enrolment.

The primary outcome of interest was the characterisation of the frequency, anatomical distribution, type, and mechanism of injuries sustained by female amateur football players during the 2023/24 season. Secondary outcomes were (1) the comparison of injury profiles between competitive matches and training sessions, and (2) the structural comparison of injury patterns in amateur female football with data from professional female football as reported in the VBG Sports Reports [8], using identical injury definitions and questionnaire instruments across both cohorts.

### 2.2. Participants and Recruitment

Within the Bavarian Football Association (BFV), the largest regional football association in Germany, senior women’s football is organised in a competitive league pyramid from the Frauen-Bayernliga to the lowest district divisions. The vast majority of players were competing in this amateur pyramid [24]. Recreational and youth (girls’) teams were not part of the target population. The 26 participating clubs thus constitute a convenience sample of competitive amateur women’s football across multiple districts and all amateur competitive divisional levels.

Eligible clubs were identified through direct contact with team coaches, club officials, and representatives of Bavarian amateur football associations. Clubs participated voluntarily. Inclusion and exclusion criteria: Included were all female players actively registered for and competing with a participating senior women’s team in the BFV competitive league system, spanning divisions from the fourth national tier (Frauen-Bayernliga) to the lowest district divisions, during the 2023/24 season. Within participating clubs, players were eligible regardless of age, playing position, or prior injury history. No further exclusion criteria were applied.

A total of 26 clubs, comprising 450 female players, were enrolled. For each club, only the first (highest-level) women’s team was monitored. All participants were thoroughly informed about the study objectives, methodology, and confidentiality procedures before participation.

### 2.3. Injury Definitions

Injury definitions and data collection procedures followed the consensus statement by Fuller et al. [25]. A relevant injury was defined as any physical complaint sustained by a player during football participation that resulted in the player missing at least one competitive match or being unable to participate in regular team training for more than three consecutive days. This time-loss definition was applied consistently throughout the study to enable direct comparison with professional football surveillance data.

### 2.4. Data Collection

Injury surveillance was conducted using a standardised injury questionnaire (29 items), available in both paper and interactive PDF formats. The questionnaire had originally been developed at the University Hospital Regensburg for the corresponding VBG-funded study of professional female football and was kindly provided to the present study group [7]. Use of the identical instrument in both cohorts ensured direct structural comparability between amateur and professional female football data.

For each injury, the following information was collected: date of injury, anatomical location, injury type (e.g., ligament injury, fracture, muscle strain), specific diagnosis where available, injury mechanism (acute trauma or overuse), activity at the time of injury (match or training), contact mechanism (non-contact, indirect contact, direct contact), analgesic use, and return-to-activity, return-to-sport and return-to-competition timelines.

Designated team physicians or study supervisors assigned to each participating club were responsible for completing and submitting questionnaires to the Study Office of the Paracelsus Medical University (PMU), Nuremberg. Reporting was conducted monthly or within an agreed interval not exceeding three months. Data were entered into a standardised Microsoft Excel database by the research team. All injury records were anonymised before analysis.

### 2.5. Statistical Analysis

This was a descriptive observational study; as no between-group comparison was planned, no a priori sample-size or power calculation was performed, and all participating clubs and players available during the study period were included. The unit of analysis throughout was the injury (time-loss event) rather than the player, in accordance with the Fuller et al. consensus; because only injury questionnaires were completed and non-injured players were not surveyed, the number of distinct injured players is not an available denominator. Descriptive statistics were computed for all injury variables. Continuous variables are presented as means with standard deviations (m ± SD); categorical variables as absolute frequencies and percentages. Principal proportions are additionally reported with 95% binomial exact (Clopper–Pearson) confidence intervals (CI). Percentage values for injury mechanism and activity context were calculated using the number of injuries with documented information as the denominator (i.e., excluding cases with missing data). Anatomical location percentages were calculated relative to the total number of anatomical locations documented (*n* = 71), as three injuries were recorded with multiple locations. Owing to incomplete reporting of individual fields, denominators differ between variables and are reported alongside each result (injury mechanism *n* = 66, activity context *n* = 55, contact mechanism *n* = 67, anatomical locations *n* = 71; age *n* = 59, height *n* = 60). As inference was based on proportions and exact categorical tests, with the only continuous variables (age and height) reported descriptively, formal testing of data normality was not required.

To test whether the distribution of injury mechanisms (non-contact, indirect contact, direct contact) differed between knee and non-knee injuries, Pearson’s chi-square test was applied; as one expected cell count was below five, the Freeman–Halton exact test was additionally computed and used as the primary result, and effect size was expressed as Cramer’s V. For mechanism knee versus non-knee Fisher’s exact test was used, with odds ratios (OR) and 95% confidence intervals (CI); a Bonferroni-corrected threshold of α = 0.0167 was applied to the three mechanism contrasts. A two-sided *p* < 0.05 was considered statistically significant. Inferential analyses were performed in Python 3 (SciPy and statsmodels).

Due to the absence of individual exposure data (training hours and match minutes per player), injury incidence rates per 1000 h of exposure could not be calculated. Comparisons with professional football data were therefore based on proportional distributions and seasonal injury burden rather than incidence rates. Structural comparability was ensured by the use of identical injury definitions and questionnaire instruments across both cohorts.

## 3. Results

Characteristics of the documented injury events are summarised in Table 1; the entire cohort was female.

### 3.1. Study Population

A total of 68 injuries were recorded across the 26 included clubs, comprising 450 female players, during the 2023/24 season. Demographic characteristics of the documented injury events are summarised in Table 1. The mean age was 26.1 ± 8.3 years, and the mean height was 167.4 ± 6.4 cm. Age and height were documented for 59 and 60 of the 68 injury events, respectively; body weight was not recorded, so the body mass index (BMI) could not be calculated. Analgesic use at the time of injury was reported by 26.9% of affected players, and 22.7% indicated current use of hormonal contraception.

### 3.2. Injury Mechanism and Activity Context

Of the 66 injuries with documented injury mechanism, 44 (66.7%; 95% CI 54.0–77.8) resulted from acute trauma and 22 (33.3%) were attributed to overuse. Two injuries could not be classified due to missing mechanistic information. (Table 2).

Of the 55 injuries with a documented activity context, 43 (78.2%; 95% CI 65.0–88.2) occurred during matches and 12 (21.8%) during training. For the remaining 13 injuries, the activity context could not be determined. Notably, all seven anterior cruciate ligament (ACL) ruptures occurred during match play.

### 3.3. Anatomical Distribution of Injuries

**Figure 1:**
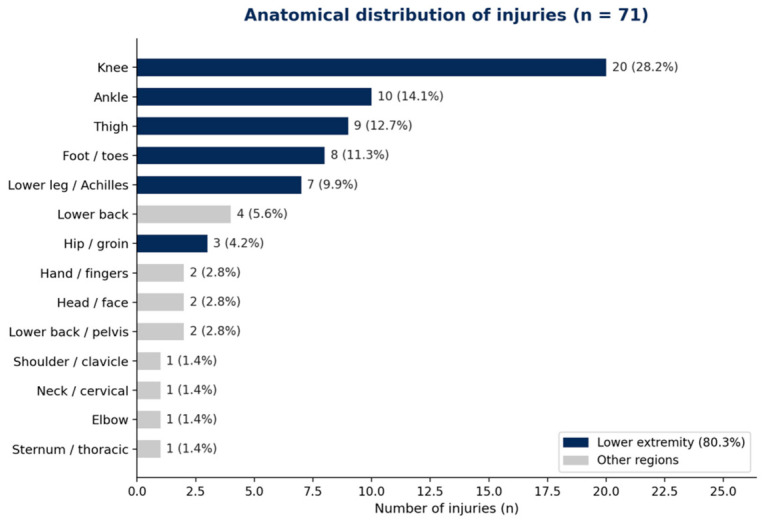
Anatomical distribution of the 68 injury events with 71 locations.

Lower-extremity injuries accounted for 80.3% of all injuries (*n* = 57 of 71; 95% CI 71.2–90.5), with the knee being the single most frequently injured region (*n* = 20; 28.2% of 71 documented locations; 95% CI 18.1–40.1). The full anatomical distribution is shown in Figure 1.

Lower-extremity locations (dark bars) accounted for 80.3% of all injuries, with the knee the single most frequently injured region (*n* = 20). Counts represent documented anatomical locations (some injuries were recorded with more than one site).

### 3.4. Injury Types and Specific Diagnoses

Ligament injuries (sprains or tears) were the most frequent injury type (*n* = 19; 27.9%; 95% CI 17.7–40.1), followed by contusions and muscle tears/strains. The complete distribution of injury types is shown in Table 3.

Among the clinically relevant structural injuries, seven ACL ruptures were recorded (10.3% of all injuries; 95% CI 4.2–20.1), all occurring during match play. Further structural diagnoses—including meniscus lesions, ankle ligament ruptures, ankle fractures, and concussions—are summarised in Table 4.

### 3.5. Injury Mechanism: Contact Versus Non-Contact

Of the 67 injuries with a documented contact mechanism, 31 (46.3%; 95% CI 34.0–58.9) were non-contact, 27 (40.3%) involved direct contact, and 9 (13.4%) involved indirect contact, corresponding to a contact-related share of 53.7% (36/67; 95% CI 41.1–66.0).

**Figure 2:**
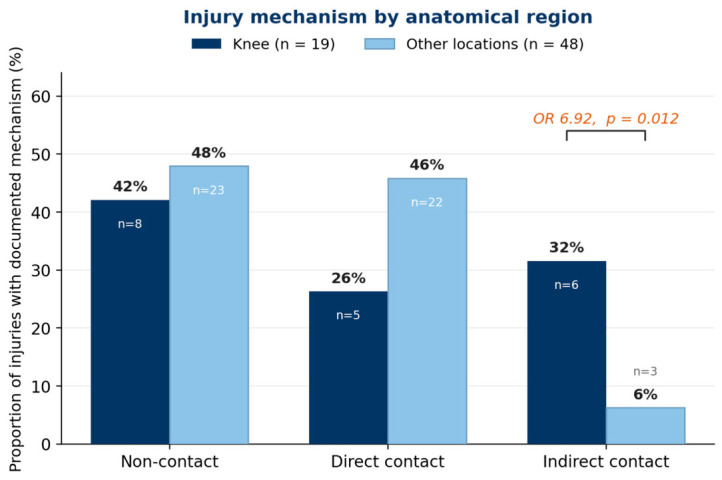
Injury mechanism by anatomical region. Indirect-contact mechanisms were markedly over-represented among knee injuries (31.6%) compared with all other locations (6.3%; OR 6.92, 95% CI 1.52–31.6; *p* = 0.012), whereas direct-contact and non-contact mechanisms were distributed similarly. Denominators are injuries with a documented mechanism (knee *n* = 19; other locations *n* = 48).

The distribution of injury mechanisms differed significantly between knee and non-knee injuries (Freeman–Halton exact *p* = 0.030; Pearson chi-square = 7.89, df = 2, *p* = 0.019; Cramér’s V = 0.34). This difference was driven exclusively by indirect-contact mechanisms, which were markedly over-represented at the knee (6/19; 31.6%) compared with non-knee locations (3/48; 6.3%) (OR = 6.92, 95% CI 1.52–31.6; *p* = 0.012). By contrast, direct-contact and non-contact mechanisms did not differ by region (*p* = 0.17 and *p* = 0.79, respectively). The mechanism distribution by anatomical region is shown in Figure 2.

Among the seven ACL ruptures, five (71%) occurred without direct contact (three indirect, two non-contact); given the small number, this is reported descriptively.

### 3.6. Return-to-Play Timelines

**Figure 3:**
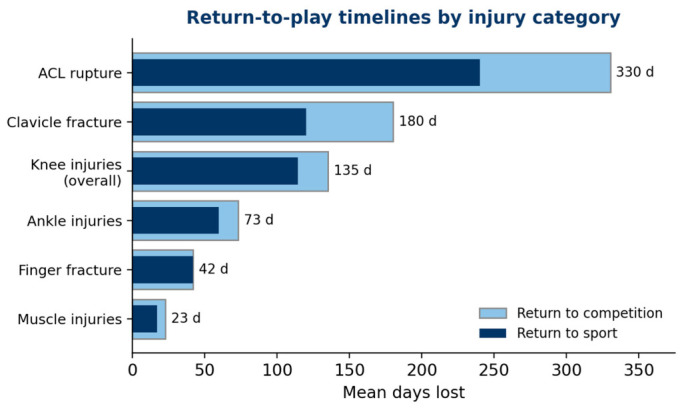
Mean return-to-play timelines by injury category. ACL ruptures entailed by far the longest absence (mean return to competition ≈ 330 days), whereas muscle injuries recovered fastest (≈23 days). Light bars = return to competition; dark bars = return to sport. Estimates are descriptive and biased towards more severe, formally tracked injuries.

Return-to-play timelines differed markedly by injury category and are presented in Figure 3.

## 4. Discussion

The most important findings of this study are that injuries in German amateur women’s football were concentrated at the knee (the single largest region, 28.2%), dominated by match rather than training exposure (78.2% vs. 21.8%), and carried a relative ACL burden (10.3%) at the upper end of professional figures, with indirect-contact mechanisms significantly over-represented at the knee. These findings support our a priori hypothesis. To our knowledge, this is the first prospective one-season cohort study to characterise the overall injury profile in German amateur women’s football specifically; prospective data from amateur women’s football elsewhere remain scarce [2].

The absolute injury burden (68 injuries among 450 players; 0.15 per player) was lower than in recent professional registries—for example, 362 injuries among 551 professional players in a single Bundesliga season [7] and a time-loss incidence of roughly 20.5 per 1000 match hours in the English Women’s Super League [6,8]. This gap most plausibly reflects structural under-reporting in the amateur setting, where injury tracking depends on coaches, volunteers, or players rather than dedicated medical staff, together with lower weekly exposure. That is why minor injuries might not be reported or included. Our figures should therefore be read as a conservative lower-bound estimate of the true amateur burden. Because the under-captured events are predominantly minor time-loss injuries, the principal findings—anatomical distribution, injury types, the match-to-training ratio, and the knee/ACL mechanisms—are unlikely to be materially affected.

The lower-extremity, knee-centred profile (knee 28.2%, ankle 15.5%, thigh 12.7%) reproduces virtually every prospective study in female football [2,26], but with an amateur-specific signature: a greater relative share of knee injuries than in professionals (28.2% vs. ~22%) and fewer thigh injuries. Within this knee burden, ACL ruptures were the clinically dominant subset (10.3% of all injuries; approximately one third of knee injuries), all sustained during match play—a share at the upper end of professional German registry and VBG data (~7–8%) [7,8,12] (interpreted with caution given the small number of events and the absence of incidence rates). This is consistent with evidence that the absolute ACL risk is higher in amateur than professional football [27] and rose further after the COVID-19 lockdown [28].

Mechanistically, indirect-contact injuries were almost confined to the knee (31.6% vs. 6.3% elsewhere; OR 6.92; *p* = 0.012), and five of seven ACL ruptures occurred without direct contact. This mirrors video-analysis data from elite women’s football, where 84% of ACL injuries occur without direct contact, the non-contact ‘pressing’ pattern predominating [16]. Because these non-contact and indirect-contact knee mechanisms are precisely those targeted by neuromuscular programmes such as the FIFA 11+, our data locate the principal modifiable injury mechanism at the amateur level, where such programmes are least implemented. The ACL findings themselves rest on only seven events and are therefore hypothesis-generating; robust inference is anchored at the level of knee injuries as a whole (*n* = 20). The wide confidence interval of this association (OR 6.92, 95% CI 1.52–31.6) further indicates that, while statistically significant, its precise magnitude is uncertain; the finding should be read as a clinically plausible direction of effect rather than a definitive estimate. Several contextual factors should further temper this interpretation: playing level, player age, training frequency and periodisation, the type of playing surface, and access to qualified medical support differ markedly between amateur and professional settings and may influence both the true occurrence of injuries and the completeness of their documentation, so the comparison with professional reference data is structural and proportional rather than causal.

Injuries clustered strongly in matches (78.2% vs. 21.8% in training)—a more pronounced imbalance than the roughly even split of professional registries [7]—reflecting lower and less periodised training exposure. Taken together, the match dominance, the acute lower-extremity trauma, and the high relative ACL burden define exactly the profile that evidence-based prevention targets: the FIFA 11+ reduces overall and lower-limb injuries by approximately 30–50% in female players when performed at least twice weekly [13,28]. Extending such low-cost neuromuscular programmes—with structured warm-ups and basic load monitoring—into the amateur game is the central practical implication of this study.

Recovery times followed the expected severity gradient (ACL rupture ≈ 330 days to competition; muscle injuries ≈ 23 days), consistent with professional data [25]. These estimates must be read with caution, because tracking depended on voluntary reporting, predominantly severe injuries were captured, inflating mean absence durations relative to cohorts in which all time-loss injuries are followed.

Several limitations apply. As a descriptive observational study, it is principally subject to reporting and recording bias (documentation by non-medical staff rather than blinded assessors), recall bias (reporting intervals of up to three months), and selection bias in the return-to-play data; minor injuries were probably under-captured. Body weight, years of football participation, playing surface, and exposure to neuromuscular prevention programmes (e.g., FIFA 11+) were not captured by the instrument; consequently, BMI could not be computed, and these variables could not be analysed. As only injured players completed questionnaires, no baseline data were available for non-injured players, precluding a whole-cohort description, an injured-versus-non-injured comparison, and any multivariable regression on player characteristics. These variables are the focus of an ongoing, dedicated study and represent priorities for future, adequately powered, exposure-based research. Individual exposure data were unavailable, precluding incidence rates per 1000 h and restricting comparisons to proportions. Finally, the number of ACL ruptures was small (*n* = 7), so ACL-specific proportions, mechanisms, and recovery times are descriptive and statistically fragile and are best read alongside larger registries [27] and video-analysis cohorts [16]. Future studies should add standardised documentation by trained staff and systematic exposure monitoring to enable incidence-based comparisons.

## 5. Conclusions

In this cohort, the amateur injury profile structurally reproduced the knee-centred, match-dominated pattern of elite female football. The relative ACL burden appeared to lie at the upper end of professional reference data, but given the small number of ACL events and the absence of incidence rates, this remains a hypothesis-generating signal that requires confirmation in larger, exposure-based studies. These findings nonetheless support extending structured neuromuscular prevention programmes into German amateur women’s football.

## Figures and Tables

**Table 1:** Characteristics of the documented injury events.

Variable	Documented Injury Events (*n* = 68)
Age, years (M ± SD)	26.1 ± 8.3
Height, cm (M ± SD)	167.4 ± 6.4
Analgesic use (%)	26.9%
Hormonal contraception (%)	22.7%

Age and height were documented for 59 and 60 of the 68 injury events, respectively; body weight was not recorded, precluding BMI calculation.

**Table 2:** Injury mechanism and activity context.

Category	*n*	%
Injury mechanism		
Acute trauma	44	66.7%
Overuse	22	33.3%
Not classifiable (missing data)	2	—
Activity context		
Match	43	78.2%
Training	12	21.8%
Not determinable (missing data)	13	—

Mechanism percentages are based on 66 classified injuries; activity-context percentages on 55 injuries with a documented context.

**Table 3:** Distribution of injury types.

Injury Type	*n*	%
Ligament injuries (sprain/tear)	19	27.9%
Contusions/bruises	13	19.1%
Muscle tears/strains	13	19.1%
Meniscus/cartilage injuries	6	8.8%
Fractures	5	7.4%
Tendon rupture/acute inflammation	4	5.9%
Other	4	5.9%
Dislocation/subluxation/joint sprain	3	4.4%
Calf muscle hardening	2	2.9%
Concussion	1	1.5%
Facial contusion	1	1.5%
Skin abrasion	1	1.5%
Gluteal injury	1	1.5%
Muscle tightness	1	1.5%
Isolated dislocation	1	1.5%
Total	75 entries	—

Percentages calculated from 68 injuries; because some injuries were assigned more than one type, the 75 type entries exceed the number of injuries.

**Table 4:** Clinically relevant structural injuries (selected severe diagnoses).

Structural Diagnosis	*n*	% of All Injuries (*n* = 68)
ACL rupture	7	10.3%
Meniscus lesion	4	5.9%
Knee ligament rupture (non-ACL)	1	1.5%
Ankle ligament rupture	5	7.4%
Ankle fracture	2	2.9%
Concussion/head contusion	2	2.9%

Percentages calculated from 68 injuries.

## Data Availability

Data is due to ethical reasons not applicable.
